# A SNP resource for studying North American moose

**DOI:** 10.12688/f1000research.13501.1

**Published:** 2018-01-10

**Authors:** Theodore S. Kalbfleisch, Brenda M. Murdoch, Timothy P. L. Smith, James D. Murdoch, Michael P. Heaton, Stephanie D. McKay

**Affiliations:** 1 Department of Biochemistry and Molecular Biology, School of Medicine, University of Louisville, Louisville, Kentucky, USA; 2 University of Idaho, Moscow, Idaho, USA; 3 U.S. Meat Animal Research Center, Clay Center, Nebraska, USA; 4 University of Vermont, Burlington, Vermont, USA

**Keywords:** Alces, moose, single nucleotide polymorphism, whole genome sequence, SNP, parentage, animal identification, genetic diversity, DNA testing, cattle genomes, sheep genomes, wildlife comparative genomics

## Abstract

**Background**: Moose ( *Alces alces*) colonized the North American continent from Asia less than 15,000 years ago, and spread across the boreal forest regions of Canada and the northern United States (US).  Contemporary populations have low genetic diversity, due either to low number of individuals in the original migration (founder effect), and/or subsequent population bottlenecks in North America.  Genetic tests based on informative single nucleotide polymorphism (SNP) markers are helpful in forensic and wildlife conservation activities, but have been difficult to develop for moose, due to the lack of a reference genome assembly and whole genome sequence (WGS) data.

**Methods**:  WGS data were generated for four individual moose from the US states of Alaska, Idaho, Wyoming, and Vermont with minimum and average genome coverage depths of 14- and 19-fold, respectively.  Cattle and sheep reference genomes were used for aligning sequence reads and identifying moose SNPs.

**Results**:  Approximately 11% and 9% of moose WGS reads aligned to cattle and sheep genomes, respectively.  The reads clustered at genomic segments, where sequence identity between these species was greater than 95%.  In these segments, average mapped read depth was approximately 19-fold.  Sets of 46,005 and 36,934 high-confidence SNPs were identified from cattle and sheep comparisons, respectively, with 773 and 552 of those having minor allele frequency of 0.5 and conserved flanking sequences in all three species.  Among the four moose, heterozygosity and allele sharing of SNP genotypes were consistent with decreasing levels of moose genetic diversity from west to east.  A minimum set of 317 SNPs, informative across all four moose, was selected as a resource for future SNP assay design.

**Conclusions**:  All SNPs and associated information are available, without restriction, to support development of SNP-based tests for animal identification, parentage determination, and estimating relatedness in North American moose.

## Introduction

*Alces alces* is the largest member of the Cervidae family, and ranges throughout the circumpolar boreal forests of Eurasia and North America ^1, 2^. The species diverged from the ancestors of domestic cattle and sheep approximately 27 million years ago ^3^. Moose are important ecologically, as a large ungulate with strong ecosystem impacts; economically, due to their value for tourism and hunting; and culturally, as a prominent symbol in many regions ^4^. Consequently, there is active management of moose populations by wildlife agencies throughout their range in North America. However, management is hampered by a lack of genetic tools for monitoring moose, assessing the genetic health of populations, and even detecting illegal harvesting. Moose populations appear to be declining in some regions, including parts of the Upper Midwest of the United States ^5, 6^, and effective management is often dependent on data that are logistically challenging and/or costly to collect.

Identifying individual animals and measuring relatedness among and within populations are important for effective wildlife management and conservation efforts ^7– 9^. Identifying individuals can be as simple as observing their unique color patterns, for example in wild dogs ( *Lycaon pictus*) ^10^. However, this is not practical in species such as moose that have few features with obvious variation between individuals. Moreover, coat color patterns provide little information about genetic relatedness. Association of younger and older animals has been used to infer relationships, for example in swift foxes ( *Vulpes velox*) where pups at a den are presumed to be offspring of the attending parents based on the monogamous behaviors they exhibit. However, detailed parentage studies have revealed multiple paternity within swift fox litters ^11^ and other fox species ^12^. Generally, genetic testing provides a more accurate assignment of parentage and supports unique identification of individuals in the vast majority of instances, as well as an estimation of intra- and inter-population genetic variability.

Genetic testing using DNA markers has been applied to human, livestock, and wildlife studies for many years ^13– 16^. This form of testing first gained popularity with the development of microsatellite short tandem repeat (STR), and mitochondrial genome markers, concurrent with the development of DNA amplification and sequencing technologies. Approximately 5 to 11 microsatellite markers from cattle, sheep, and caribou ( *Rangifer tarandus*) have been adapted for moose studies ^17– 22^. These studies form the basis of our current understanding of North American moose population structure and genetic diversity. DNA technology developments in the past decade have led to the replacement of microsatellite and mitochondrial genome markers with SNP markers because SNPs are more abundant, have greater stability over generations, are more accurately genotyped, and are amenable to automating the genotyping processes ^23^. Moreover, panels of SNPs broaden the use of genotyping for management and conservation efforts, because they can provide not only identification of individuals and parentage, but also estimation of inbreeding and relatedness, and detection of admixture between populations of wildlife. For example, an SNP-based approach has been used for conservation efforts in endangered species such as the Iberian lynx ( *Lynx pardinus*) ^24^ and Tasmanian devil ( *Sarcophilus harrisii*) ^25^, as well as more common but wide-ranging species like the brown bear ( *Ursus arctos*) ^26^. The application of SNP-based approaches, however, requires first the identification of polymorphisms segregating in the populations being studied, and developing assays that support accurate genotyping. Accordingly, a SNP panel spanning the genome of North American moose would be useful for addressing fundamental questions about population genetics in this species.

Low genetic diversity among North American moose populations has been previously reported ^17, 18^, making development of SNP panels challenging. The low diversity has been attributed in the prevailing theory, to a relatively recent (ca. 11,000–14,000 years ago) colonization from Asia and subsequent founder effect induced by extended range expansion from an original small group of animals ^27^. However, no definitive evidence has been presented that refutes an alternative hypothesis, that North American moose experienced a severe population bottleneck at some time in the past ^20^, as occurred for North American bison ( *Bison bison*) populations ^28^. Whether a founder effect, bottleneck, or both, the small effective population size simultaneously increases the need for developing genetic tools for management and the challenge of creating SNP marker panels.

Discovery of SNPs in a species has generally been preceded by development of its reference genome assembly. Using this assembly, whole genome sequence (WGS) reads from individual animals can be aligned, and differences between segregating alleles identified. However, creation of a reference assembly still represents a significant barrier for most research communities interested in wildlife species. Fortunately, an alternative approach that uses the reference genomes of related species has been developed ^29^, and shown to effectively identify high-confidence SNPs likely to be segregating within the target species. Here we report the whole genome sequencing of four moose genomes, each obtained from distant geographic regions of North America, and the use of the cattle and sheep reference genomes to align the sequence data and identify SNPs likely to be segregating among moose populations. A set of criteria was developed to select the potentially most useful set of moose SNPs, and to identify 317 autosomal variants meeting these criteria. The associated sequence information was made freely available, and represents a resource for developing genotyping assays to support moose genetic research.

## Methods

### Ethical statement

This article contains no studies performed with animal subjects, and thus, no additional institutional ethical permits were required. Samples for DNA extraction were donated by private individuals not associated with this research. These were hunters that had legally harvested moose during the firearm hunting season in their state. No additional approvals were needed, since all hunters obtained valid hunting licenses for the harvesting of moose.

### Animal samples

**Figure 1.**
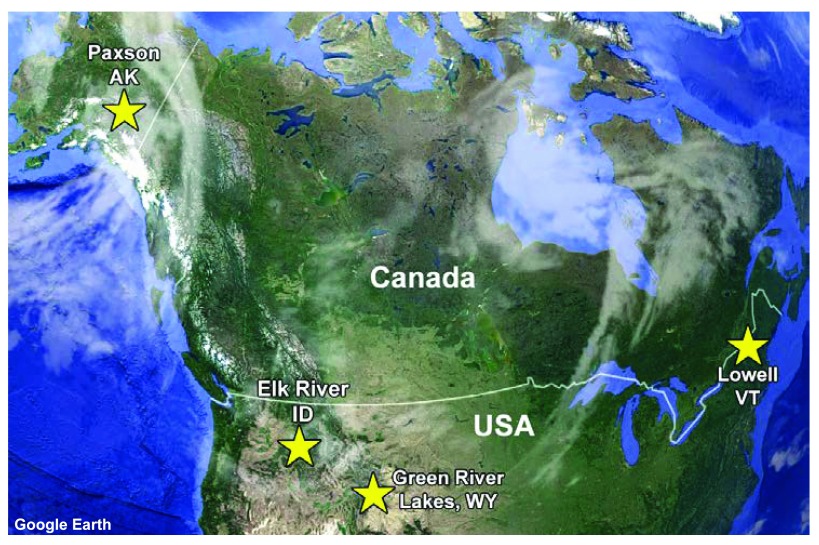
Locations in North America where the moose used in this study were collected.

Samples of muscle tissue were obtained from four animals likely comprising three putative subspecies of *A. alces* based on their location in North America: *A. gigas*, *A. shirasi*, and *A. americana* ^30^. These animals were harvested at four distinct geographic locations ( Figure 1) and entered as BioSamples in NCBI BioProject Accession PRJNA325061 ( Table 1). As is typical, hunters removed the internal organs in the field, the carcasses were chilled, and the meat was subsequently processed for frozen storage. Each of the four owners donated approximately 50 g of frozen tissue from their harvested animal, and that tissue was archived at USMARC for use in this project.

**Table 1.** Whole genome sequence information and alignment statistics for four North American moose.

								Reads mapped (%)	
Species	Animal identifier	BioSample number	Location or breed	Sex	Estimated aligned read depth ^a^	Gb sequence collected	Total reads (millions)	Bt_UMD3.1	Oar_v3.1
*Alces alces* *gigas*	HM2013 (201524011)	7695254 ^b^	Paxson, Alaska, USA	M	19.2	64.1	461	13.0	10.4
*Alces alces* *shirasi*	JC2001 (200124009)	7695255 ^b^	Green River Lakes, Wyoming, USA	F	13.7	45.7	327	13.5	10.8
*Alces alces* *americana*	R199	7695256 ^b^	Lowell, Vermont, USA	M	23.2	77.3	653	8.8	6.8
*Alces alces* *shirasi*	Clearwater06	7695257 ^b^	Elk River, Idaho, USA	M	20.0	66.8	549	8.9	6.9
*Bos taurus*	19969811	5216015 ^c^	USA Hereford	M	14.2	47.5	314	88.4	nd ^d^
*Ovis aries*	199735001	5216748 ^e^	USA Rambouillet	M	13.7	42.7	319	22.2	83.8

Key: ^a^Based on the observed linear relationship between aligned read depth (y) and total gigabases (Gb) of genomic sequence collected (x) with a quality score ≥ to 20, where y = 0.3x in cattle and sheep genomes ^38, 39^.

^b^NCBI BioProject number 325061

^c^NCBI BioProject number 324822

^d^Not determined

^e^NCBI BioProject number 324837

### WGS production, alignment, and SNP genotyping

DNA was extracted from muscle with a typical phenol:chloroform method and stored at 4°C in 10 mM TrisCl, 1 mM EDTA (pH 8.0) as previously described ^31^. Approximately 5 μg of moose genomic DNA was fragmented by focused-ultrasonication to generate fragments less than 800 bp long (Covaris, Inc. Woburn, Massachusetts USA). These fragments were used to make an indexed, 500 bp paired-end library according to the manufacturer’s instructions (TruSeq DNA PCR-Free LT Library Preparation Kits A and B, Illumina, Inc., San Diego, California USA). After construction, indexed libraries were pooled with other indexed samples in groups of four to eight, and sequenced with a massively parallel sequencing machine and high-output kits (NextSeq500, two by 150 paired-end reads, Illumina Inc.). After sequencing, the raw reads were filtered to remove adaptor sequences, contaminating dimer sequences, and low-quality reads. Pooled libraries with compatible indexes were repeatedly sequenced until a minimum of 40 Gb of sequence with greater than Q20 quality was collected for each animal. Previous results showed that this level of coverage provided genotype scoring rates and accuracies that exceeded 99% ^29^.

The DNA sequence alignment process was similar to that previously reported ^29^. Briefly, FASTQ files corresponding to a minimum of 40 Gb of Q20 sequence were aggregated for each animal. The reference assemblies for both UMD3.1 ^32^ and Oar_v3.1 were downloaded from the NCBI genomes download site and indexed for use with the Burrows Wheeler aligner (BWA) version 0.7.12 ^33^. The fastq files corresponding to R1 and R2 runs for the paired end libraries of each respective animal were aligned individually using the BWA aln algorithm and bovine reference assembly UMD3.1. The R1 and R2 datasets were then merged and collated using BWA sampe. The process was repeated for the mapping of the reads to the ovine Oar_v3.1 reference assembly. The resulting sequence alignment map (SAM) files were converted to binary alignment map (BAM) files, and subsequently sorted using Samtools (version 0.1.18) ^34^. PCR duplicates were marked in the BAM files using the Genome Analysis Toolkit (GATK, version 1.5-32-g2761da9) ^35^. Regions in the mapped dataset that would benefit from realignment due to small insertions and deletions were identified using the GATK module RealignerTargetCreator, and realigned using the module IndelRealigner. The BAM file produced at each of these steps was indexed using Samtools. The resulting indexed BAM files were made available via the Intrepid Bioinformatics genome browser http://www.intrepidbio.com/, with groups of animals linked at the USMARC WGS browser ( mapped to cattle, mapped to sheep). The raw reads were deposited at NCBI BioProject Accession PRJNA325061. Some SNP variants were identified manually by inspecting the target sequence with Integrative Genomics Viewer (IGV) software version 2.1.28 ^36, 37^, as described in previously ^38^. In these cases, read depth, allele count, allele position in the read, and quality score were taken into account when the manual genotype determination was made.

### Variant detection and filtering

The above mapping efforts produced BAM files for the alignments to both UMD3.1, and Oar_v3.1. The BAM files for all four animals were analyzed simultaneously for variation against both the UMD3.1 and the Oar_v3.1 genomes. The GATK UnifiedGenotyper was used with the genotype mode (-gt_mode) flag set to DISCOVERY, and the likelihood model (-glm) flag was set to BOTH in order to identify both single nucleotide variants, and small insertions and deletions. The maximum number of alternate alleles (--max_alternate_alleles) flag was set to allow only three. Other than those mentioned, default parameters were used. Samtools was used to generate a pileup file containing the measured allele and depth of coverage at each position for all four animals. Variant sites in the four moose were filtered for having a minimal read depth of ten, and a minimum genotype quality score of 30. The SNPs were filtered for having a minor allele frequency (MAF) of 0.5, with both homozygous genotypes present among four animals. Fifty bases of flanking DNA sequence on either side of the targeted moose SNP were analyzed for nucleotide alleles that were homozygous in all four moose yet different from the cattle or sheep reference sequences. These nucleotide sites were flagged as potential moose “species-specific” alleles and the 101 bp of context sequence was edited to create a moose consensus reference sequence. The 101 bp of moose consensus sequence derived from the alignment of one reference genome was then tested for alignment to the other reference genome. Moose SNPs with MAFs of 0.5, and having been derived independently from alignment to both reference genomes, were manually assigned genome-wide bins based on their chromosome and proximity as inferred by alignment with the cattle genome. The goal of assigning markers to bins was to minimize linkage while allowing automated SNP assay design software the opportunity to select the best candidate marker for each distinct genomic region. All of these conservative filters were intended to maximize marker informativity in North American moose populations, and minimize potential technical difficulties with SNP assay designs that rely on oligonucleotide hybridization for genotype detection.

## Results

An average of 63.5 Gb total genome sequence was collected for four moose. Based on similar estimates in cattle and sheep, this would correspond to an average read depth of 19-fold coverage if aligned to a moose reference genome of similar quality ( Table 1). However, when cattle and sheep reference genomes were used, an average of 11.0 and 8.7% of the moose reads were aligned, respectively. For comparison, the same alignment method was performed with sets of bovine and ovine genomic sequences and resulted in 88.4% and 83.8% reads aligned to their respective genome assemblies ( Table 1). For cross-species comparison, 22.2% of the ovine set of genomic sequence reads were aligned to the bovine assembly. Although the moose read depth was low when averaged across the entire genome of cattle or sheep, at conserved genome regions it was consistent with the expected average read depth of 19-fold. Thus, the moose read depth in conserved genomic regions appeared to be sufficient for identifying polymorphic sites and accurately assigning variant alleles.

**Figure 2.**
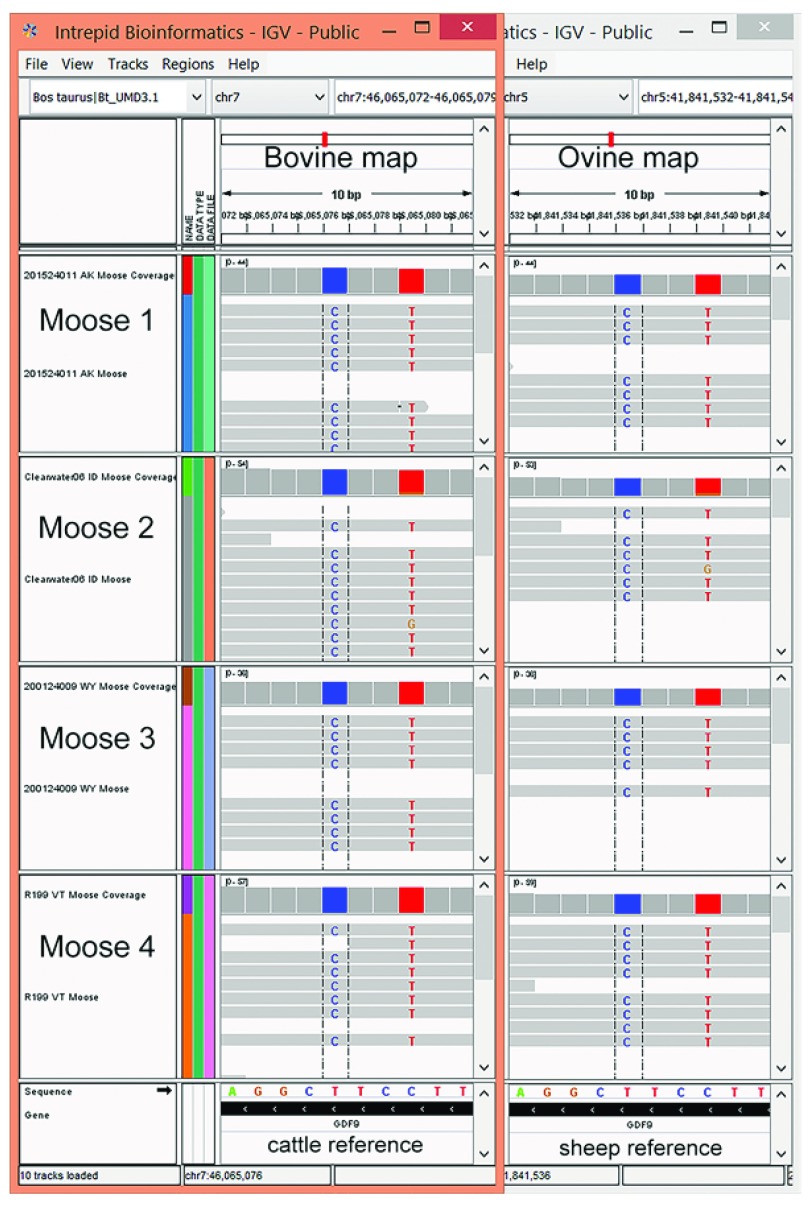
Computer screen images of species-associated nucleotide differences in moose. Overlapping computer screen images of moose WGS data aligned to bovine and ovine reference genomes, respectively, showing two moose-associated nucleotides in the *GDF9* gene. The bovine UMD3.1 and ovine Oar_v3.1 map positions for the variant sites are chr7:46,065,076 - 46,065,079 and chr5: 41,841,536 - 41,841,536,539, respectively.

**Figure 3.**
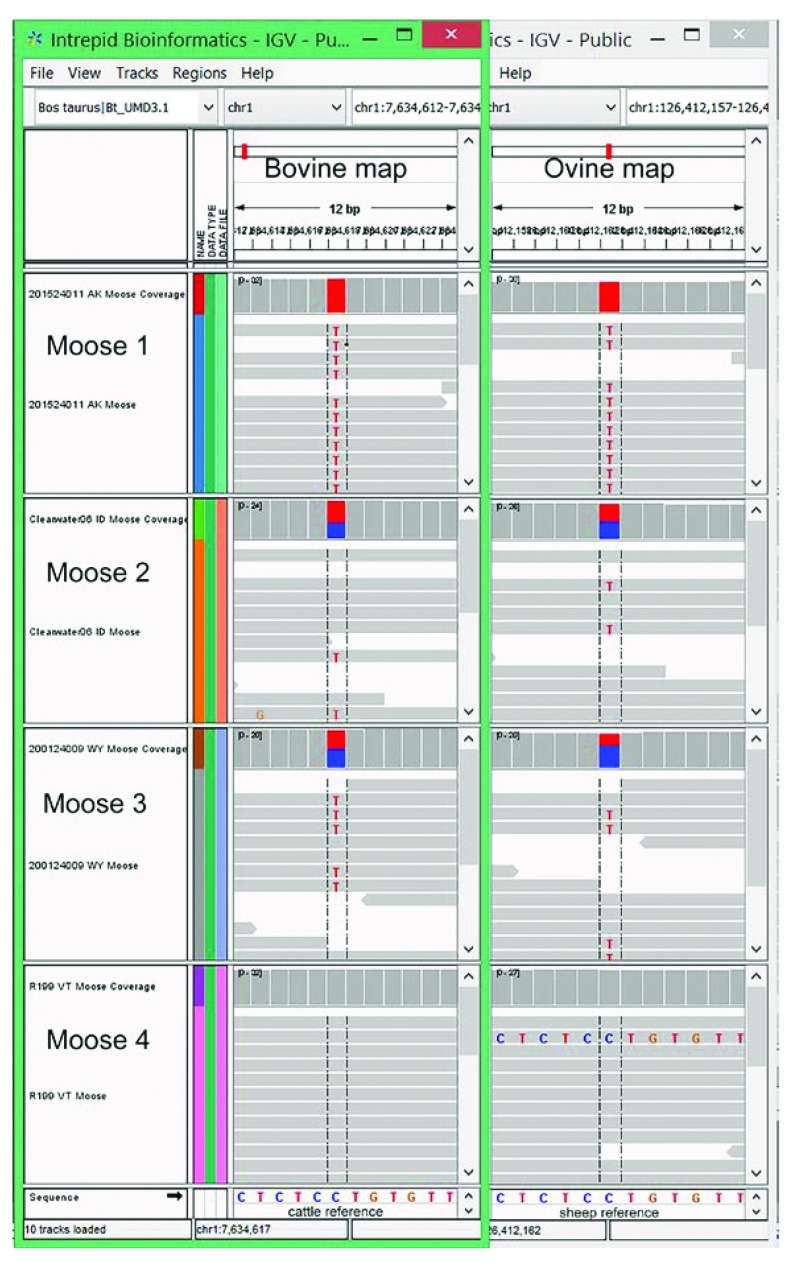
Computer screen images of a highly informative moose SNP. Overlapping computer screen images of moose WGS data aligned to bovine and ovine reference genomes, respectively, showing a highly informative SNP. Screenshots from IGV software showing one of 317 moose SNPs with a 0.5 MAF, both homozygous genotypes present, and aligned to genomic regions conserved in all three species. The bovine UMD3.1 and ovine Oar_v3.1 map positions are chr1:7,634,617 and chr1: 126,412,162, respectively.

Alignment of moose reads to the cattle and sheep genomes identified approximately 48.3 million and 39.7 million sites that differed from the reference assemblies, respectively. These included SNPs, insertions and deletions, and sites where moose-associated nucleotide differences occurred. The latter sites were defined as having homozygous genotypes in the four moose, with alleles differing from those in cattle or sheep ( Figure 2). After stringent filtering for read depth and alignment quality, there were 1,095,371 and 813,006 moose variants identified with the respective cattle and sheep genome assemblies ( Table 2). Approximately 96% of these were homozygous moose-associated nucleotide differences ( Supplementary file S1 and Supplementary file S2). The remaining 46,005 and 36,934 variants were moose SNPs identified by the respective cattle and sheep alignments ( Supplementary file S3 and Supplementary file S4). The MAF distribution of the moose SNPs was similar for both sets with the large group having a 0.125 MAF (approximately 37%, Table 2). The most informative moose SNPs (i.e., “highly informative”) were defined as those with a 0.5 MAF and both homozygous genotypes present among any of the four moose, and are candidate SNPs that may have arisen to a high MAF prior to the species arrival in North America ( Figure 3). There were 1,341 and 1,014 of these moose SNPs identified with the cattle and sheep alignments, respectively ( Table 2).

**Table 2.** North American moose SNPs identified by aligning WGS to bovine and ovine reference genomes.

		Reference assembly	
Step	Progressively filtered variant sets	Bt_UMD3.1	Oar_v3.1
1	Variants passing depth and quality filters ^a^	1,095,371	813,006
2	Moose-associated nucleotide differences ^b^	1,049,080	775,700
2	SNPs	46,005	36,934
3	SNPs with 0.125 MAF	16,815	13,698
3	SNPs with 0.250 MAF	12,502	10,106
3	SNPs with 0.375 MAF	13,369	10,710
3	SNPs with 0.500 MAF	3,319	2,420
4	SNPs with 0.500 MAF and both homozygotes present	1,341	1,014
5	SNPs with conserved flanking regions ^c^	773	552
6	SNPS with independent alignment to both reference genomes ^d^	317	317

Key: ^a^Autosomal chromosome alignment with minimum read depth of ten and minimum genotyping quality score of 30. There were approximately 60,600 and 58,200 additional moose SNPs in the respective UMD3.1 and Oar_v3.1 alignments that were heterozygous in all four moose. However, these were excluded from the SNP counts because this artifact is caused by sequence read misalignment.

^b^These sites are difference from the reference and homozygous in all four moose.

^c^101 bp flanking regions of SNPs with 0.5 MAF that could be unambiguously identified by BLAT alignment to the other reference assembly ( Table S1 and Table S2). These regions also contained no SNPs among the four moose.

^d^SNP that were independently identified by alignment in each reference genome and manually grouped into 216 chromosomal bins for assay design ( Table S3).

Candidate moose SNPs were further excluded when the flanking sequences in one reference genome were not uniquely identified in the other. This left 773 and 552 highly informative moose SNPs identified in conserved regions of the cattle and sheep genomes, respectively ( Supplementary Table S1 and Supplementary Table S2). Of these 1,325 highly informative SNPs, 1,008 were unique between the two sets, while 317 were common to both sets. The latter represents the most informative moose SNPs, with the highest flanking sequence conservation, due to their independent alignment to both reference genomes ( Table 2).

**Figure 4.**
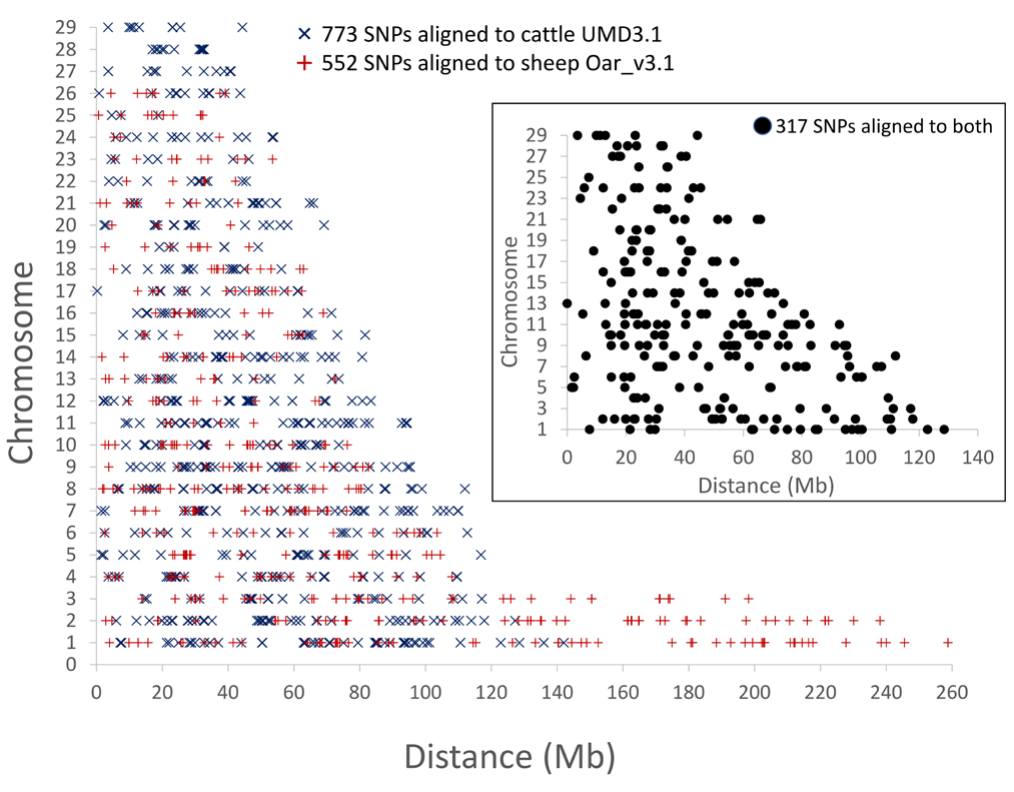
Genome-wide distribution of highly informative moose SNPs. The distribution of moose SNPs with 0.5 MAF relative to the cattle and sheep chromosomal locations (see Table S1 and Table S2 for marker details). The inset shows the chromosomal map positions with the cattle UMD3.1 reference assembly.

The alignment coordinates of the 1,325 highly informative SNPs were analyzed for genome-wide distribution patterns that may indicate ascertainment biases caused by the variant selection. Overall, the distribution of SNP sites in the sets with 773, 552, and the 317 intersecting markers, appeared to be widespread in the cattle and sheep genomes and generally appropriate for genome-wide estimates ( Figure 4). However, some SNP clustering was observed as the set of 317 had a mean and median spacing of 5.3 and 2.1 Mb, respectively ( Supplementary Figure S1A). To facilitate SNP genotype assay design, the clustered SNPs were manually grouped into 216 bins with a mean size of approximately 8.1 Mb (median 5.9 Mb, Supplementary Figure S1B). Thus, SNP assay designs could be directed to each bin, with the option to use any SNP from that bin for multiplex assay design ( Table S3).

**Figure 5.**
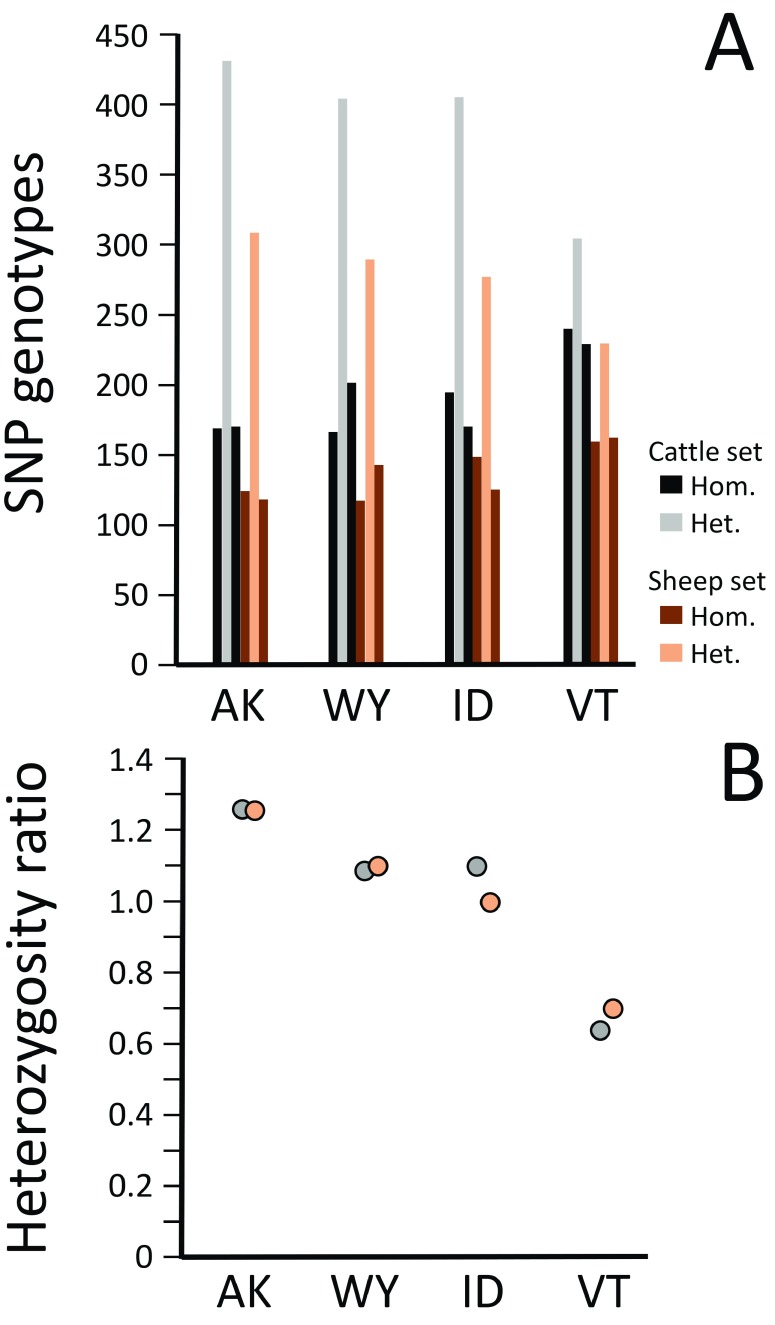
Heterozygosity analysis of moose genomes. The ratio of heterozygous to homozygous genotypes from four moose was evaluated with sets of 773 and 552 markers SNPs with 0.5 MAF (see Table S1 and Table S2 for marker details). ( **A**) Genotype counts for each of the four animals with the 773 moose SNPs identified in the alignment to cattle ( Table S1) and the 552 moose SNPs identified in the alignment to sheep ( Table S2). ( **B**) The heterozygosity ratios calculated for each of the four animals from the 773 SNP set (grey circles); and the 552 SNP set (tan circles). The ratio consisted of the number of heterozygous sites divided by the combined number of homozygous sites.

Genotype analysis for the 773 and 552 moose SNPs derived from the cattle and sheep alignments, respectively, showed that each moose had approximately the same proportion of opposing homozygous genotypes ( Figure 5A, Supplementary Table S4 and Supplementary Table S5). However, there were significant differences in the ratio of heterozygous genotypes to homozygous genotypes ( Figure 5B). The Alaskan moose had the most favorable average heterozygosity ratio (1.26), followed by the moose from Wyoming and Idaho (1.10 and 1.06, respectively), and the Vermont moose (0.68). Note that the numerical value of the ratios calculated from these SNP is likely an underestimate of the within-animal genome-wide heterozygosity, because there may be ascertainment bias resulting from targeting of SNP discovery to genomic regions conserved between three species. The SNP allele sharing between each of the four moose was analyzed with the sets of 773 and 552 markers to obtain a genome-wide measurement of their relatedness. This was possible because the method for selecting each of these SNPs was not dependent on which two of the four moose were heterozygous. The pair of moose from Alaska and Idaho had the highest proportion of shared alleles (0.430 and 0.397), while the Alaska and Vermont pair had the lowest (0.255 and 0.279, Table 3). Together, the genotype results with these sets of 773 and 552 SNPs indicate that there was a west-to-east pattern of decreasing genetic diversity in the four moose used in this study.

**Table 3.** Proportion of heterozygous sites shared between pairs of moose from Alaska (AK), Idaho (ID), Wyoming (WY), and Vermont (VT), USA.

	Proportion							
	SNPs used from cattle alignment (n = 773)				SNPs used from sheep alignment (n = 552)			
Source	AK	ID	WY	VT	AK	ID	WY	VT
AK	1.000	-	-	-	1.000	-	-	-
ID	0.430	1.000	-	-	0.397	1.000	-	-
WY	0.378	0.323	1.000	-	0.391	0.317	1.000	-
VT	0.255	0.268	0.325	1.000	0.279	0.281	0.320	1.000

The combined set of 1,008 highly informative moose SNPs were also evaluated for their relative proximity to genes in the annotated reference assemblies of cattle and sheep. In the sets of 773 and 552 moose SNPs, 256 and 181 were present within genes, respectively ( Table S6 and Table S7). Some genes contained more than one polymorphism, and thus, there were 221 and 178 total cattle and sheep genes, respectively, with highly informative SNPs. Of these genes with moose SNPs, 84 were identified in both cattle and sheep alignments. In addition, there were a number of informative SNPs in noteworthy genes that did not pass the read depth and quality score filters. For example, the prion gene ( *PRNP*) affects susceptibility to spongiform encephalopathies such as chronic wasting disease in cervids. By manually viewing the *PRNP* coding sequence with IGV software, a coding SNP with 0.5 MAF and both homozygotes present was identified (M217I, Table 4). Thus, the publicly searchable and viewable moose WGS presented here represents a novel genomics resource that may facilitate candidate gene-based research in this species.

**Table 4.** Moose gene variants identified by viewing selected genes in IGV.

Bovine UMD3.1				Moose genotype ^a^				Allele frequency	
BTA	Position	Gene	Feature	AK	WY	VT	ID	A1	A2
5	45,830,842 ^b^	*IFNG*	Intron 1	G ^c^	S	C	S	0.50	0.50
13	47,415,079 ^b^	*PRNP*	CDS, M217I ^d^	K	K	G	T	0.50	0.50
2	6,587,679	*ANKAR*	Intron 2	W	A	W	W	0.63	0.38
2	6,219,619	*MSTN*	Exon 5	G	C	S	C	0.63	0.38
3	22,970,184	*PDE4DIP*	Exon 16	T	T	C	Y	0.63	0.38
5	66,599,950	*IGF1*	CDS, I27V ^e^	C	T	Y	T	0.63	0.38
7	22,883,135	*ICAM1*	Intron 1	Y	Y	C	Y	0.63	0.38
8	78,244,133	*UBQLN1*	Exon 3	Y	Y	T	T	0.75	0.25
16	27,304,417	*TLR5*	Exon 2	C	G	G	S	0.63	0.38
26	20,694,701	*DNMBP*	Exon 17	W	W	A	W	0.63	0.38

Key: ^a^Based on samples from four individuals sourced from Alaska (AK), Wyoming (WY), Vermont (VT), and Idaho (ID), USA.

^b^Highly-informative moose parentage SNPs with 0.5 MAF and both homozygous genotypes present among the four moose.

^c^Homozygotes are denoted with the one-letter nucleotide code. Heterozygotes are denoted with IUPAC/IUBMB ambiguity codes: R = a/g, Y = c/t, M = a/c, K = g/t, S = c/g, W = a/t. ^40^.

^d^The *PRNP* codon number 217 refers to the number system in cattle. In moose, this codon is at position 209.

^e^The *IGF1* codon is in exon 2 and the numbering for codon 27 is the same in cattle as in moose.

## Discussion

We sequenced four moose from regions that span the United States, to approximately 19-fold genome coverage, and aligned them to the cattle and sheep reference genomes. Approximately 10% of moose sequences were aligned and used to identify more than 40 k moose SNPs in this cross-species approach. The relatively low alignment rate may be a reflection of the 27 million year average molecular divergence time between moose and non-cervid members of the Pecora infraorder ^3^. In spite of the alignment rate, 1,008 highly informative moose SNPs were identified for future use in developing DNA-based genetic tests to support forensic and wildlife conservation activities. These 1,008 moose SNPs were derived from the intersection of two overlapping sets aligned to cattle (773 SNPs) and sheep (552 SNPs) reference genome assemblies. The 1,008 moose SNPs were refined to a minimal subset of 317 moose SNPs found in the most highly conserved genome regions. All of these markers are publicly available and ready for validation on a variety of SNP genotyping technology platforms. An important first step in evaluating these SNPs will be characterizing their MAFs in wild populations of North American moose. The online whole genome moose sequences, together with reference genotypes ( Supplementary Table S4 and Supplementary Table S5) and DNA from these four moose, provide the opportunity for immediate design, testing, and validation of these candidate parentage SNPs.

Genotype information from the 1,008 moose SNPs was useful for measuring genome-wide differences in DNA sequence diversity among the four individuals. Measurements of heterozygosity and allele sharing showed that the Alaskan moose was the most diverse, the Vermont moose was the least, with the moose from Idaho and Wyoming being intermediate. This is consistent with a species that crossed the Bering Land Bridge into Alaska and radiated outward from west to east across North America. SNPs have been previously used to estimate genome diversity in other species with low genetic diversity like the European bison ( *B. bonasus*) ^41^ and the Tasmanian devil ( *S. harrisii*) ^25^. A caveat with our results is the overall heterozygosity of each moose may be underestimated due to ascertainment bias for highly informative SNPs in highly conserved genomic regions. In other words, variation in conserved moose genome regions may occur at a lower rate than that in non-conserved regions. In spite of this potential ascertainment bias, the results suggest that combinations of these markers may be useful in detecting population structure.

An important unanswered question is: how informative will these SNPs be in moose populations? Population-wide data to address this question will require development and application of genotyping assays, and assembly of pertinent samples for testing, which was beyond the scope and resources of the present report. The data presented here, which identify polymorphisms with alternate homozygous genotypes in a limited sample of only four individuals, suggest that the SNP selected represent variation that existed prior to arrival of moose in North America.

## Conclusions

These moose SNPs and associated sequence information are available for use without restriction, and provide a basis for developing commercial SNP-based “parentage” SNP DNA tests for validation in North American moose populations.

## Data availability

FASTQ files for the four moose combined are available in NCBI SRA, with contiguous accession numbers SRX3218250 - SRX3218281.

The SRA accession numbers for each individual are:

SRX3218264 - SRX3218271, Alaska moose HM2013;

SRX3218254 - SRX3218259 and SRX3218262 - SRX3218263, Wyoming moose JC2001; SRX3218250 - SRX3218253 and SRX3218272 - SRX3218275, Vermont moose R199; and SRX3218260 - SRX3218261 and SRX3218276 - SRX3218281, Idaho moose Clearwater06.

The data are part of NCBI BioProject Accession PRJNA325061.

In addition, access to the aligned sequences is available via the USDA: http://www.ars.usda.gov/Research/docs.htm?docid=25590 (moose aligned to cattle), and http://www.ars.usda.gov/Research/docs.htm?docid=25712 (moose aligned to sheep).

Download access to the BAM files is available at the Intrepid Bioinformatics sites: http://server1.intrepidbio.com/FeatureBrowser/customlist/record?listid=7919250313 (moose aligned to cattle), and http://server1.intrepidbio.com/FeatureBrowser/customlist/record?listid=7919250315 (moose aligned to sheep).

## Supplementary materials

**Table S1.** List of 773 moose parentage SNPs aligned to bovine reference assembly UMD3.1.

**Table S2.** List of 552 moose parentage SNPs aligned to ovine reference assembly Oar_v3.1.

**Table S3.** List of 317 candidate moose parentage SNPs grouped into 216 bins for use in multiplex assay design.

**Table S4.** Genotypes for 773 moose SNPs aligned to the bovine reference assembly UMD3.1.

**Table S5.** Genotypes for 552 moose SNPs aligned to the ovine reference assembly Oar_v3.1.

**Table S6.** List of 256 moose parentage SNPs occurring within genes annotated in bovine reference assembly UMD3.1.

**Table S7.** List of 181 moose parentage SNPs occurring within genes annotated in ovine reference assembly Oar_v3.1.

**Figure S1.** Distributions for 317 moose parentage SNPs and their 216 marker groups.

**File S1.** VCF file with 1,095,371 moose-specific variants aligned to the cattle UMD3.1 reference assembly.

**File S2.** VCF file with 813,006 moose-specific variants aligned to the sheep Oar_v3.1 reference assembly.

**File S3.** VCF file with 46,005 moose SNPs aligned to the cattle UMD3.1 reference assembly.

**File S4.** VCF file with 36,934 moose SNPs aligned to the sheep Oar_v3.1 reference assembly.
