# Supplementary material for: A SNP resource for studying North American moose
Source: F1000Res. 2018 Jan 10;7:40. [Version 1] doi: 10.12688/f1000research.13501.1 (PMC5801567; doi:10.12688/f1000research.13501.1)
Supplement: Supplementary file 8 [file f1000research-7-14659-s0007.tgz › a9e581fa-ad97-42b1-b193-9450262e7661.pdf]

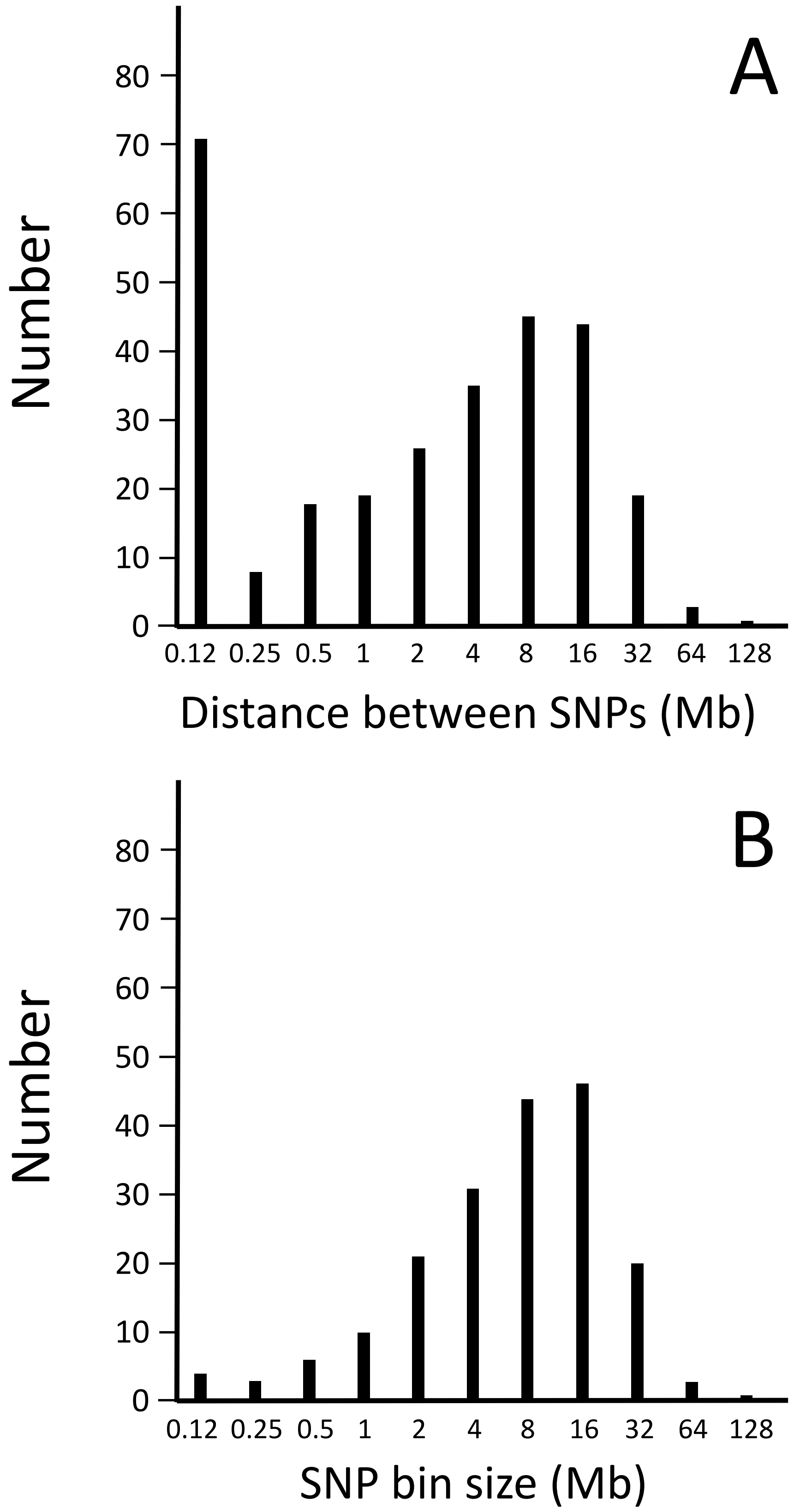

Figure S1. Distributions for 317 moose parentage SNPs and their 216 marker groups. (**A**) Distribution of inter-SNP distances for 317 moose SNPs aligned to the bovine genome reference assembly UMD3.1. (**B**) Distribution of bin sizes for 216 bins containing 317 SNPs manually assigned to bins based on their proximity.
