# Supplementary material for: A SNP resource for studying North American moose
Source: F1000Res. 2018 Jan 10;7:40. [Version 1] doi: 10.12688/f1000research.13501.1 (PMC5801567; doi:10.12688/f1000research.13501.1)
Supplement: Peer review history

# Peer review: Referee response for version 1

**DOI:** 10.5256/f1000research.14659.r29739

**Published:** 2018-02-06

**Authors:** Kris J. Hundertmark

**Affiliations:** 1 Department of Biology and Wildlife, Institute of Arctic Biology, University of Alaska Fairbanks, Fairbanks, AK, USA

This study presents SNP discovery and variation of moose in North America, which are very valuable data. First, moose are an understudied species and their genetics can tell us a lot about the phylogeography of species after the LGM. As the authors state, moose in North America have generally low neutral nuclear diversity, and are less diverse than their European counterparts (information from Siberia, if it exists, has not been published in English). They are also less diverse than many northern species and many species of cervids, such as caribou, white-tailed deer, and elk. Hypotheses have been proposed for these observations and have been tested with mtDNA, but only recently have nuclear markers been used to study phylogeography in European moose. No such tests have been conducted in North America. This study provides new tools to spur moose research, although I agree with Reviewer 2 that microsatellites and mtDNA still have their uses.

 The inclusion of 4 individuals in this study is good, as is the broad geographic range of the samples across North America. The methods are appropriate, although as the first reviewer points out, RADSeq could have been used in SNP discovery and has some advantages over the methods used, but I believe that this report on moose is secondary to the authors’ overall goals and we are fortunate that Kalbfleish et al. decided to pursue this and share their findings. The comparison to reference genomes, although from bovids not very closely related to moose, yielded some advantages, including identifying SNPs in important functional genes, such as the PRNP locus. Nonetheless, a white-tailed deer genome was made public in summer 2017 and although these authors may not be willing to start over by comparison of their data to a very similar genome (same subfamily and same number of chromosomes) the prospect exists and should be pursued. I can see the point of Reviewer 2 concerning Fig. 4 and assumed synteny but I believe I got the intended message from the figure as is. But I think mentioning somewhere that North American moose have 34 pairs of autosomes when discussing the success of mapping SNPs to the reference genomes would be appropriate.  

 I find the last sentence interesting in that the authors believe the SNP variants they found existed prior to moose entering North America. Considering that event likely happened within the last 15,000 years that is a very reasonable statement, and may cause some to think that SNPs may contain little information about geographic variation in moose and all that goes with it. Given the morphological and behavioral differences between, say, Alaskan moose and those in the eastern continent, however, it is obvious that moose have evolved rapidly in that time, despite limited genetic diversity due to Pleistocene bottlenecks and founder effects. How that translates to current SNP diversity and what the latter may be able to tell us about moose are exciting questions to be asked, for which this manuscript sets the stage.

 Minor comments:

 2 ^nd^ paragraph of Introduction: should be “among individuals” not “between individuals.”

 Third paragraph of Introduction: should be “a SNP-based approach” not “an SNP-based approach.”

 Last paragraph of Introduction, last sentence: should it be “has been made freely available” rather than “was made freely available?”

 Last paragraph of WGS Production …, last words of 2 ^nd^-to-last sentence: Should be “previously.” not “in previously.”

 Discussion, last sentence: should this be “SNPs” instead of SNP?”

I have read this submission. I believe that I have an appropriate level of expertise to confirm that it is of an acceptable scientific standard.

# Peer review: Referee response for version 1

**DOI:** 10.5256/f1000research.14659.r30045

**Published:** 2018-01-30

**Authors:** Paul Stothard

**Affiliations:** 1 Department of Agricultural, Food and Nutritional Science, University of Alberta, Edmonton, AB, Canada

This well-written manuscript describes the discovery and characterization of single nucleotide polymorphisms (SNPs) in moose. These SNPs will be valuable for future research and conservation efforts, as they can be used for animal identification, assigning parentage, and estimating intra- and inter-population genetic variability. The SNP discovery and genotyping are done using well-established and clearly described approaches, and care has been taken to avoid false positive SNPs (requiring that both homozygous genotypes be observed for example). The raw sequencing data is available through the SRA database, while the aligned reads (BAM) files are available via the USDA and Intrepid Bioinformatics sites. The final filtered variants are provided with flanking sequence in the supplementary materials. Larger, less-filtered collections of variants are provided in the form of VCF files, again in the supplementary materials. The clear, detailed manuscript and the raw data and progressively filtered results will make is easy for others to reproduce or make use of the results of this work.

 Minor comments

 1. In the last paragraph of the introduction the authors note the challenge of creating a whole genome assembly for use in SNP discovery, and that the use of an existing reference genome from a related species can be an effective alternative. In the discussion section the authors mention that the cross-species mapping approach, however, has the drawback of targeting conserved regions of the genome. I am curious as to why the authors chose not to employ a technique like RADseq for SNP discovery, as it does not depend on the availability of a reference genome and would allow them to better assess minor allele frequency, through the inclusion of more individuals. Also, RADseq would be equally effective at targeting conserved and non-conserved regions. The reasons for not using RADseq (or its potential value in future studies) could be addressed in the introduction.

 2. The figure legends for Figure 2 and Figure 3 could be expanded slightly to explain to readers not familiar with IGV which elements represent reads, coverage, reference sequence. Also, in Figure 3 it isn't clear to me why the sequence of one read is shown (moose 4, second read from top, aligned to the sheep reference).

 3. In the Methods section "fastq" is written as "FASTQ" and "fastq".

I have read this submission. I believe that I have an appropriate level of expertise to confirm that it is of an acceptable scientific standard.

# Peer review: Referee response for version 1

**DOI:** 10.5256/f1000research.14659.r30007

**Published:** 2018-01-30

**Authors:** Joshua M. Miller

**Affiliations:** 1 Université du Québec à Montréal, Montreal, Quebec, Canada; 2 University of Alberta, Edmonton, Canada

In this work Kalbfleisch *et al.* develop a novel set of SNP loci for moose. To do so the authors generated genomic sequence data from 4 moose across their range in the United States, aligned the reads to both cow and sheep reference genomes, and then applied a series of filters to select a subset of loci in highly conserved regions. The resulting set of loci showed a gradient of diversity decreasing from west to east, consistent with hypothesized colonization. The authors state that these loci will serve as a resource for future management and conservation applications. I think that this study has laudable goals and adds a valuable resource for moose conservation. However, there are some issues the analyses and presentation that need to be clarified.

 Overall comments

- The alignment and filtering procedures seem overly restrictive. Authors note that only ~10% of their reads aligned to the cow and sheep reference genomes (not surprising given the levels of divergence among the species) meaning that 90% of the data was essentially thrown away. Why not start with a *de novo* assembly of the moose sequence?
  - Along these lines there are several recent papers that detail a hybrid procedure for genome construction that begins with *de novo* assembly and then apply cross-species alignment for scaffolding, e.g. https://bmcbioinformatics.biomedcentral.com/articles/10.1186/s12859-017-1911-6
- If part of the goals is to eventually use these loci for “population genetic applications” (e.g. looking for population structure and assigning individuals to those populations) it strikes me that selecting loci that are in such heavily conserved regions may result in biased estimates. Can the authors comment on this? Doesn’t this procedure result in a lot of “moose specific” variants being missed?
- I can understand that it is not the objective of this paper to create a draft genome sequence for the moose (though the authors note in several places that this would be useful, and the data generated here seem appropriate to attempt this), but then more justification as to why this was not attempted needs to be given.
- Did the authors check and make sure that the “highly conserved genomic regions” are not in repetitive elements? A blast search of these regions should do the trick.

 

 Specific comments

- In the sentence in the introduction starting with “DNA technology developments…” saying that SNPs have “replaced” microsatellites and mitochondrial DNA is a gross overstatement. There are many studies still using these markers and there are many applications where these markers may be preferable. Would be better to say something along the lines that SNPs have gained in use.
- As currently written, the sentence in the introduction starting with “Moreover, panels pf SNPs broaden…” is incorrect. Microsatellites and mitochondrial DNA also provide means of measuring inbreeding and relatedness among individuals. This should be rephrased to state that SNPs may be better at these estimates, likely due to the factors listed in the previous sentence (abundance, accuracy, etc.)
- I would couch the statement that previous estimates of low genetic diversity in moose will make it hard to discover SNPs. This may be true, but it has not been tested and estimates of diversity from 5-11 microsatellites likely will not reflect genome-wide patterns.
- Remove “in the prevailing theory,”
- I find it striking that in the final paragraph of the introduction there is no mention of the plethora of genotype-by-sequencing approaches that are used for SNP discovery and population genetic analyses but do not require a reference genome, e.g Peterson *et al. *(2012) ^1^.  This should be addressed.
- When you state the sub-species names in the Methods refer readers to Table 1 so that they know which occurs where relative to your samples.
- Which program or programs were used to filter the raw reads?
- State what “UMD3.1” and “Oar_v3.1” are when you first introduce them in the methods
- Is binning relative to the cow genome actually informative for moose? Are the karyotypes comparable? How many chromosomes does the moose have?
- Along this same line, I think having the variants locations for both UMD3.1 and Oar_v3.1 plotted on top of each other in Figure 4 is misleading. It suggests that the locations are syntenic, which is not true. For instance, the plot implies that no variants were discovered on chromosomes 27, 28, and 29 in the sheep genome, when really this is impossible as sheep only have 27 autosomes. I would suggest re-doing this figure with separate panels for each figure, though see the caveat above.
- In Figure 5A why are there two bars for each of the “Hom” counts but only one for the “Het”?

I have read this submission. I believe that I have an appropriate level of expertise to confirm that it is of an acceptable scientific standard, however I have significant reservations, as outlined above.

## References

[1] PetersonBKWeberJNKayEHFisherHSHoekstraHE: Double digest RADseq: an inexpensive method for de novo SNP discovery and genotyping in model and non-model species. *PLoS One*.2012;7(5) : 10.1371/journal.pone.0037135 e37135 10.1371/journal.pone.0037135 22675423PMC3365034
